# Characterizing the Microenvironment of Cerebral Arteriovenous Malformations to Test Novel Treatment Modalities

**DOI:** 10.3390/brainsci15111145

**Published:** 2025-10-25

**Authors:** Kavin Wazhi, Fred C. Lam, Santosh Guru, Yusuke S. Hori, Deyaldeen AbuReesh, Lorelei Shoemaker, David J. Park, Steven D. Chang

**Affiliations:** Department of Neurosurgery, Stanford University School of Medicine, Stanford, CA 94305, USA; kavin_wazhi@brown.edu (K.W.); fredlam@stanford.edu (F.C.L.); sg928@cam.ac.uk (S.G.); yshori@stanford.edu (Y.S.H.); abureesh@stanford.edu (D.A.); lshoe@stanford.edu (L.S.); djpark@stanford.edu (D.J.P.)

**Keywords:** brain arteriovenous malformations, radiosurgery, embolization, neurosurgery, organoids, animal models

## Abstract

Brain arteriovenous malformations (bAVMs) consist of a tangled nidus of abnormal dilated vessels characterized by direct connections between arteries and veins that lack an intervening capillary bed, creating a high-to-low flow pressure system that is predisposed to spontaneous hemorrhage with significant associated neurologic morbidity and mortality. Treatment options for bAVMs include the following: surgical resection, intravascular embolization to obliterate blood flow through the AVM, and radiosurgery. Understanding the molecular mechanisms of bAVM formation and factors that predispose it to hemorrhage can lead to novel treatments that can improve the prognosis for patients. This review summarizes emerging insights into the complex and dynamic molecular mechanisms of bAVMs. Dysregulation in key VEGF, TGF-β/BMP9/10–ENG–ALK1–SMAD4, Notch, and MAPK/ERK signaling pathways drive abnormal angiogenesis in both syndromic and sporadic forms, with KRAS/BRAF/MAPK21 mutations specifically linked to the latter. Advances in bAVM-induced animal models have corroborated many of the genetic profiles found in humans, and they continue to provide novel insights into bAVM mechanisms. Collectively, these mechanistic findings are guiding translational advances, with targeted therapies and liquid biopsy approaches emerging as avenues for precision treatment and improved patient outcomes.

## 1. Introduction

Brain arteriovenous malformations (bAVMs) are rare arteriovenous shunts, resulting from abnormal connections between cerebral arteries and veins that form a central nidus [1]. The prevalence of bAVMs is estimated to be approximately 10 to 18 per 100,000 adults [2,3]. The most common symptomatic manifestations of bAVM are intracranial hemorrhages followed closely by seizures; 11% of intracranial hemorrhages have a one-month case fatality while there is an approximate 8% risk of seizures five years post-diagnosis [1]. These risk factors make it critical to efficiently and effectively treat bAVMs. Current standard of care options to treat bAVMs include the following: microsurgery, embolization, and stereotactic radiosurgery (SRS) [4]. Each of these treatment options has its own series of risks and benefits in terms of patient experience and post-treatment outcomes. Microsurgery ensures a high chance of bAVM obliteration coupled with low post-op hemorrhage occurrence with rates of 96% and 0.18% per 100 person-years, respectively [1]. However, microsurgery mandates an open craniotomy, requiring the bAVM to be in an area with an appropriate access corridor; microsurgery is also typically associated with longer hospital stays and recovery. The alternative option of vascular embolization provides far more flexibility in its use by combining various treatment modalities to achieve safer obliteration in certain cases. Lone embolization results in a lower rate of obliteration at 13% per 100 person-years, with neurological complications and hemorrhages occurring at rates of 6.6% and 1.7% per 100 person-years, respectively [1]. SRS, on the whole, tends to garner results in between that of microsurgery and embolization, with an obliteration rate and hemorrhage rate of 38% per 100 person-years and 1.7%, respectively [5]. A benefit of SRS is its minimal invasiveness and convenience for patients, but there is a latency period where the bAVM is not obliterated post-SRS, resulting in hemorrhage risk. Given these accumulated risks, there is an unmet need to gain a deeper understanding of the interactions within the microenvironment of bAVMs that predispose bAVMs to hemorrhage and rebleeding, as a means to develop strategies to reduce rupture risk.

Brain AVMs were traditionally thought to be congenital lesions arising from aberrant vasculogenesis during the fourth and eighth weeks of gestation. However, there have been few reported cases of fetal bAVMs, and with noted recurrence after surgical resection and remodeling over time [6,7], as well as reports of post-traumatic [8] and post-infectious bAVMs [9]. These observations suggest more dynamic and complex mechanisms at play. Similarly, there exists a “second hit” hypothesis, in which patients develop bAVMs after cerebral aneurysm occlusion, SRS treatments, hemorrhagic strokes, the growth of brain tumors, or encephalitic demyelinating lesions, further exemplifying the dynamic interactions between the local microenvironment of the brain that trigger bAVM formation [10]. We herein review the current understanding of the molecular biology driving bAVM formation, the signaling pathways and genes that are affected that lead to bAVM hemorrhage, and the novel therapeutic avenues of research that intend to decrease hemorrhage risk.

## 2. The Physiology of Brain Arteriovenous Malformation Formation

### 2.1. Angiogenesis

A recently published single-cell atlas, comparing normal human brain vasculature compared to surgically resected human bAVMs, reported differential gene expression across 15 major cell populations [11]. Similarly to previously published gene signatures, the study identified enriched vascular cell signatures, including the following: endothelial cells (*CLDN5*), pericytes (*KCNJ8*), smooth muscle cells (*MYH11*), and perivascular fibroblasts (*DCN*). The study also identified differential gene expression signatures to the following four arteriovenous segments: arteries, capillaries, venues, and veins. Brain AVM endothelial cells (ECs) were enriched in *TXNIP*, a regulator of glucose metabolism and oxidative stress [12], reflecting the increased metabolic state of bAVM arterial endothelial cells. Other genes that were enriched in human endothelial AVM zonations included *VEGF* in the arteries, *MFSD2A* in the capillaries, and *ACKR1* in the veins [13,14,15]. Taken together, this study identified distinct, conserved clusters of genes in the endothelial arteriovenous zonations in human bAVMs.

Data from animal and human studies support dysregulated angiogenesis as playing a role in the formation of bAVMs. Human bAVM ECs have upregulation of the VEGF and TGFβ pathways, and increased EC turnover. The localized injection of adeno-associated viral vector expressing *VEGF* (*AAV-VEGF*) into the brains of adult transgenic mice deficient in *Eng* or *Acvrl* induced focal angiogenesis, while the embryonic deletion of *Eng* caused postnatal formation of brain, spinal cord, and intestinal AVMs [16]. Endoglin expression was found in the endothelium and adventitial layer of arteries and arterioles, with expression in the mesenchymal cells of the adventitia and perivascular connective tissue in the arterialized veins of sporadic human bAVMs [17]. However, unlike hereditary hemorrhagic telangiectasia (HHT) type 1 bAVMs, in which endoglin expression is reduced, levels of endoglin were found to be normal with increased numbers of endoglin-positive endothelial and adventitial cells. Endoglin expression was also seen in fibroblasts in the perivascular stroma, suggesting an active role in vascular remodeling in response to increased blood flow and shear stress [17]. In addition, an intronic variant of *ACVRL-1*, IVS3-35A>G has been found to be associated with bAVMs [18,19], further implicating the TGFβ pathway in the physiology of bAVM formation.

### 2.2. Pathophysiology of Syndromic-Related Brain Arteriovenous Malformations

#### 2.2.1. Hereditary Hemorrhagic Telangiectasias

**Figure 1:**
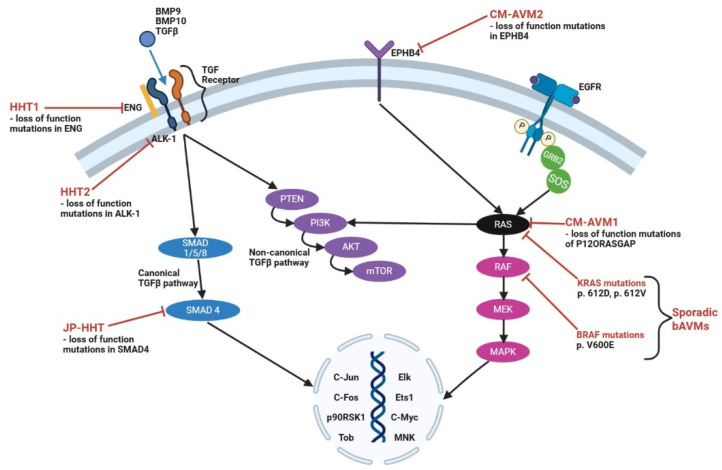
Signaling pathways involved in the formation of sporadic and syndromic brain AVMs. Abbreviations: BMP9 = bone morphogenic protein 9; BMP10 = bone morphogenic protein 10; TGFβ = transforming growth factor beta; ENG = endoglin; TGF = transforming growth factor; HHT1, HHT2 = hereditary hemorrhagic telangiectasia type 1, type 2; ALK-1 = activin A receptor-like type 1; SMAD 1, 4, 5, 8 = mothers against decapentaplegic homolog 1, 4, 5, 8; JP-HHT = juvenile polyposis/hereditary hemorrhagic telangiectasia; PTEN = phosphatase and tensin homolog; PI3K = phosphatidylinositol-3-kinase; AKT = protein kinase B; mTOR = mammalian target of rapamycin; C-Jun = transcription factor Jun; C-Fos = protein C-Fos; p90RSK1 = 90 kilodalton protein ribosomal protein S6 kinase 1; Tob = Tob protein; Elk = transcription factor Elk; Ets1 = transcription factor Ets1; C-Myc = proto-oncogene c-Myc; MNK = mitogen activated protein kinase-interacting kinases; CV-AVM1, 2 = capillary malformation-arteriovenous malformation types 1 and 2; EPHB4 = Ephrin B4; EGFR = epidermal growth factor receptor; GRB2 = growth factor receptor-bound protein 2; SOS = son of sevenless; RAS = RAS protein; RAF = RAF protein; MEK = mitogen-activated protein kinase kinase; MAPK = mitogen activated protein kinase; P120RASGAP = p120 RAS guanosine triphosphatase activating protein; KRAS = Kirsten rat sarcoma virus oncogene; BRAF = B-Raf proto-oncogene; and bAVMs = brain arteriovenous malformations.

The majority of bAVMs are sporadic, with only approximately 5% of patients associated with genetic syndromes, such as hereditary hemorrhagic telangiectasia (HHT) and/or capillary malformations-arteriovenous malformations (CM-AVM) [20]. HHT (also known as Osler–Weber–Rendu syndrome) is characterized by mucocutaneous telangiectasias and AVMs. However, the familial germline mutations in the genes underlying these syndromes have shed important insights into the signaling pathways and networks that contribute to the pathophysiology of bAVM formation. These families of genes include the following: transforming growth factor-β (*TGF-β*), endoglin (*ENG*), activin receptor-like kinase (*ALK1*), *SMAD*, *KRAS*, and *MAPK* [20], and are involved in the regulation of angiogenesis [21] (Figure 1). Approximately 5–20% of HHT patients have at least one bAVM, with multiple bAVMs being a predictive factor of HHT [22,23,24]. HHT-associated bAMVs tend to have a smaller nidus than sporadic bAVMs, with no statistical significance in age at diagnosis, prevalence of intracranial hemorrhage (ICH), or age at first ICH [25]. There are three types of HHT, characterized by mutations in members of the TGFβ/BMP signaling pathway: (1) type 1 results from loss-of-function (LoF) mutations in one copy of endoglin (*ENG*) [26,27]; (2) type 2 results from LoF mutations in activin A receptor like type 1 (*ACVRL1* or *ALK1+/−*) [28]; and (3) mutations in *SMAD4* cause a combined syndrome of juvenile polyposis and HHT (JP-HHT), accounting for 2% of HTT cases [29] (Figure 1).

#### 2.2.2. Wyburn–Mason Syndrome

Wyburn–Mason syndrome (WMS), also known as Bonnet–Dechaume–Blanc syndrome, is a rare non-hereditary congenital neurocutaneous disorder characterized by AVMs. WMS lesions typically affect the skin, retina, and brain; the resulting bAVMs tend to be ipsilateral, with the midbrain most affected [30]. An embryonic defect is thought to result in the spread of vascular lesions that involve both the developing optic cup and anterior neural tube [30,31,32]. The specific genetic and molecular mechanisms underpinning WMS are not yet understood, and current clinical practices in treating associated bAVMs resembles that of sporadic bAVMs [30,31].

#### 2.2.3. Sturge–Weber Syndrome

The presence of bAVM in patients with Sturge–Weber syndrome (SWS) are limited to a few exceptional cases [33]. SWS typically presents as facial cutaneous vascular malformations (port-wine stains), with the presence of port-wine stains underlying greater risk in ocular and neurological disorders [34,35]. Brain involvement in SWS patients is typically marked with leptomeningeal vascular malformation [36]. Recurrent somatic mosaic of the R183Q mutation in GNAQ was found to be the major determining factor in the development of SWS [34,36]. The R183Q GNAQ mutation is thought to hyperactivate the downstream Ras/Raf/MEK/ERK and mTOR pathways, resulting in SWS [36]. Increases in levels of Angiopoietin-2 from the GNAQ mutation have been implicated in the formation of capillary malformations associated with SWS [37]. Despite our growing understanding of the mechanisms contributing to SWS, the mechanistic link between bAVM occurrence with SWS remains a mystery. One paper has pointed to the theory that the R183Q-GNAQ mutation may disrupt arteriovenous specification through Notch signaling in the formation of malformed episcleral vasculature, but direct links to bAVMs remain speculative [37].

### 2.3. Pathophysiology of Sporadic Brain Arteriovenous Malformations

Much of the pathophysiology of bAVM formation can be derived from understanding the pathways that drive the normal development of the vascular system. Fibroblast growth factor (FGF) drives the differentiation of mesodermal progenitors into hemangioblasts, which are thought to be the precursor cells of endothelial precursor cells (EPCs) and hematopoietic stem cells (HSCs) [38,39]. The differentiation of EPCs into ECs is an essential step in vasculogenesis. EPCs and HSCs form blood islands under vascular endothelial growth factor (VEGF) signaling [40]. Angiogenesis (the formation of new blood vessels from pre-existing vessels), is also under the influence of VEGF signaling. Sprouting angiogenesis requires proteolysis of laminin and type IV collagen in endothelial basement membranes, a process that is initiated by matrix metalloproteinases (MMPs), heparanases, cathepsins, and the urokinase plasminogen activator [41,42,43]. The formation of a mature, organized, vascular network depends on the inhibitory signaling via plasminogen activator inhibitor-1 (PAI-1) and the tissue inhibitors of metalloproteinases (TIMPs) [44], which lead to the release of angiogenic factors such as VEGF, FGF, and chemokines, and anti-angiogenic molecules to complete angiogenesis [45]. Human and animal studies support the role of dysregulated angiogenesis as involving the TGFβ and VEGF pathways [46]. Further dysregulation of cell proliferation and endothelial cell migration also leads to elevated angiogenic signaling [47].

An early hypothesis explaining the sporadic formation of bAVMs postulates that a hypoxic event occurs in the surrounding brain tissues, leading to the upregulation and over-expression of VEGF [48]. This hypothesis is supported by the findings that VEGF is over-expressed in tissues adjacent to bAVMs [49,50,51] and in peripheral blood samples [52,53]. Hypoxia inducible factor 1 (HIF-1) is normally undetectable in oxygenated tissues; however, its expression is increased in hypoxic conditions providing a feedback loop leading to the increased expression of VEGF and its receptors [48]. Another condition that leads to the concomitant upregulation of HIF-1 and VEGF is intracranial venous hypertension [54,55], which has been shown to induce high expression of VEGF in blood vessel endothelium in rodents [56]. The upregulation of VEGF creates a positive feedback loop leading to the increased expression of metalloproteinases MMP-2 and MMP-9 in bAVMs, and of cerebral cavernous malformations [57,58]. The glycoprotein carcinoembryonic antigen-related cell adhesion molecule 1, which is involved in angiogenesis and cell proliferation, has been previously linked to bAVM rupture in male patients [59].

Abnormally elevated levels of inflammatory and immune cells have also been associated in both unruptured and ruptured bAVMs, including perinidal macrophages [60], neutrophils, and T lymphocytes [61]. Cx3cr1+ microglia and Ccr2+ macrophages are present in bAVMs in *Alk1* knockout mice [62]. Similarly, astrocytes and endothelial cells upregulate the expression of glutamate transporter 1 (GLUT1) in AVM nidus vessels compared to control vessels [63]. Whole exome sequencing of endothelial cells from resected bAVMs has shown them to harbor activating somatic *KRAS* G12D or G12V, *BRAF*, and *MAP2K1/MEK* mutations [64,65,66,67,68]. The upregulation of MEK/ERK activity can also be detected in bAVM tissues that do not harbor KRAS mutations, suggesting the central roles of mitogenic pathways in the pathophysiology of sporadic bAVM formation [64] (Figure 1).

While activating somatic mutations in *KRAS*, *BRAF*, and *MAP2K1* have been implicated in aberrant MAPK signaling in bAVM pathogenesis, studies also suggest the involvement of broader dysregulation of endothelial developmental programs. Human bAVM tissue has been found to aberrantly express genes implicated in venous and lymphatic specification, such as *COUP-TFII*, *PROX1*, *SOX18* that were co-expressed in PECAM+ ECs which correlated with upregulated cellular proliferation [69]. The mixed venous/lymphatic identity of the bAVM endothelium implies that, in its development, the bAVM undergoes a reprogramming of endothelial identity [69]. This reprogramming of endothelial identity observed in bAVMs may only reflect one part of a broader endothelial plasticity. Novel research suggests that endothelial–mesenchymal transition (End-MT) signaling may play a role in bAVM pathophysiology. A study by Shoemaker and colleagues reported that compared to normal brain tissue, bAVM tissue expressed End-MT associated transcription factors like KLF4, SNAI1, and SNAI2 along with key mesenchymal markers Vimentin, ACTA2, and S100A4 at much greater levels [70]. Additionally, the strong collagen deposition and high expression of PAI-1 throughout the bAVM tissue further implicates the process of End-MT in vessel remodeling and lesion maintenance [70]. Collectively, Shoemaker’s studies reveal that dysregulated endothelial identity and plasticity, rather than genetic mutation alone, could underlie the maladaptive remodeling characteristic of bAVMs. We have summarized the genes involved in the formation of bAVMs in Table 1 for our readers.

The recent single-cell analysis of human bAVM samples by Winkler and colleagues further exemplifies the diverse cellular make-up of bAVMs, identifying spatially distinct CLDN5+ endothelial cells, TAGLN+ smooth muscle cells, CCL19+ fibromyocytes, and COL1A2+ perivascular fibroblasts [11]. Clusters of myeloid cells, vessel-associated microglia, dendritic cells, perivascular macrophages, monocytes, CD4+ and CD8+ T cells, regulatory T cells, B cells, and natural killer cells were also found to be infiltrated into the perivascular space and adjacent brain that was surrounding the AVMs. Vessel-associated CD11c+ antigen-presenting cells, IBA1+P2RY12- macrophages, and IBA1+P2RY12+ microglia were also found infiltrated into the bAVM vasculature. Finally, AIF1+P2RY12- monocytes were over-represented in ruptured AVMs, suggesting the upregulation of the immune system associated with AVM hemorrhage. Taken together, these studies reveal an intricate and dynamic cellular microenvironment that is associated with bAVMs, which helps to remodel the surrounding brain milieu following bAVM rupture. We have summarized these cell types for our readers in Table 2.

### 2.4. Pathophysiology of Acquired Brain Arteriovenous Malformations

The hypothesis that bAVMs can be acquired comes from isolated reports of de novo cases [8,89], recurrences in pediatric patients after surgical resections [90,91,92], and remodeling throughout the post-resection follow-up period [93,94]. Lasjaunais and colleagues postulated that bAVMs were an indirect sequelae of other intracranial pathologies leading to remodeling of the capillarovenous junction to form secondary acquired bAVMs [95]. Similarly, patients who acquired bAVMs after stereotactic radiotherapy treatments, hemorrhagic and ischemic stroke, traumatic brain injury, cerebral aneurysm occlusion, brain tumors, or demyelinating encephalitic lesions, are thought to develop bAVMs via a “second hit” phenomenon [10].

## 3. Animal Models of Brain AVMs

Studies have shown that adenovirus Cre recombinase transgenic mice with heterozygous and homozygous *Alk1* or *Eng* mutations show different vascular phenotypes. In the brains of *Eng*-floxed mice, the homozygous deletion of *Eng* led to a severe vascular dysplasia phenotype in mice treated with VEGF compared to heterozygous *Eng* mice [96]. Inducible *Eng-* or *Alk1*-conditional knockouts, specifically in brain ECs but not in pericytes or macrophages, led to the formation of bAVMs in adult mice, suggesting that there is a cell-specific lineage in bAVM pathogenesis [16,97]. Interestingly, *Eng* or *Alk1* mutations in a small portion of ECs and in bone-marrow-derived ECs were also sufficient to cause bAMV formation, confirming a central role for ECs in bAVM pathogenesis [96,98,99]. Kim and colleagues demonstrated that the over-expression of *Alk1* can rescue the AVM phenotypes in *Alk1-* and *Eng*-inducible knock out mice via normalizing the expression of the *Notch* and *Smad* pathway genes in *Eng*-deficient ECs [100]. Activation of Notch1 and Notch4 in ECs of mouse brains induced bAVM formation [78,101]. Inhibition of Notch signaling via the deletion of the recombination signal binding protein for immunoglobulin kappa J region (*Rbpj*) in ECs of postnatal mice also led to bAVM formation [102], while *Alk1* knockout mice have endogenously decreased Notch signaling, suggesting an intimate connection between *Alk1* and *Notch* involvement in vasculogenesis [103]. The over-expression of soluble ENG has been shown to cause bAVM formation in mice. Soluble ENG binds to bone morphogenic protein type 9 (BMP9), inhibiting blood vessel formation and causing bAVM inflammation [104,105]. Inhibiting BMP9 and BMP10 induces AVMs in the retina [106], and studies suggest that BMP9 and BMP10 are likely natural ligands for the ENG/ALK1 signaling pathway [107], thereby suggesting that the BMP9/10-ENG-ALK1-SMAD4 pathway plays a role in bAVM formation in HHT patients [108,109].

Sporadic bAVMs have been formed in mice by generating EC-specific *Kras* G12D or G12V gain-of-function mutations [71], while the adenovirus-associated viral vector infection of *Kras* G12D into the brain ECs of mice also promotes the development of bAVMs via the activation of the mitogen-activated protein kinase (MAPK) pathway [110]. In the same study in which Fish and colleagues were able to generate bAVM in mice by introducing EC-specific *Kras* G12D or G12V mutations, they also generated bAVM using the same technique in zebrafish and were able to show that zebrafish embryos harboring the EC-specific *Kras* G12V mutation had a higher incidence of cranial hemorrhage, which was not seen in the embryos expressing wild-type Kras [71]. This correlates well with the findings of somatic mutations in members of the KRAS/MAPK pathways in human sporadic bAVM and peripheral AVM samples [64,66,67,68,77,111].

Murphy and colleagues expressed constitutively active *int3*, the murine homolog of Notch4, in ECs of tetracycline-regulated transgenic mice [78]. Mutant mice died between 2 and 5 weeks of age, with signs of neurological dysfunction including ataxia and seizures, which were evident in approximately 25% of the mice. The histology of the mutant brains demonstrated visual evidence of intracranial hemorrhage occurring most often in the cerebellum, followed by the neocortex, but never in the brainstem. Underlying the areas of hemorrhage were enlarged and tangled vessels, resembling bAVMs. Leveraging their Tet-on/off transgenic system to repress endothelial *int3* expression, the authors were able to reverse the neurological deficits in postnatal day 20- or day 21-old mice, further supporting the role of Notch expression in the pathophysiology of bAVMs [78]. We have summarized current animal models of bAVMs in Table 3.

## 4. Targeted Therapeutic Approaches for the Treatment of AVMs

**Figure 2:**
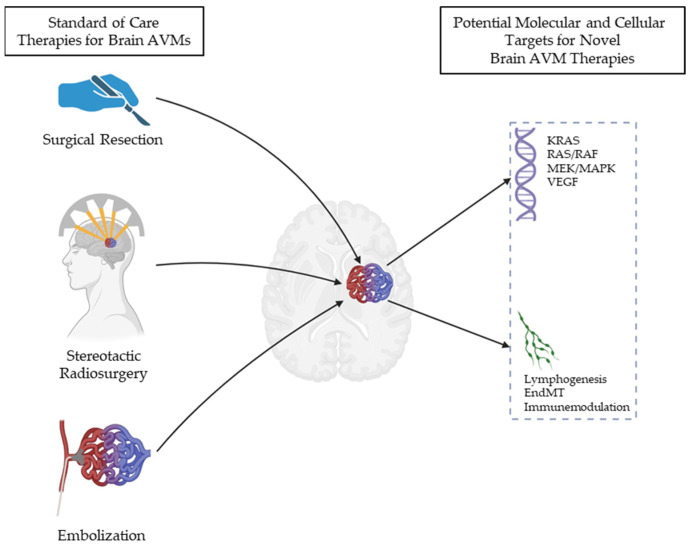
Schematic of the standard of care and potential novel targets for brain AVM therapies. Abbreviations: KRAS = Kirsten rat sarcoma virus oncogene; RAF = RAF protein; MEK = mitogen-activated protein kinase kinase; MAPK = mitogen activated protein kinase; and AVMs = arteriovenous malformations.

Brain AVMs are currently treated with surgery, radiosurgery, or embolization (Figure 2). Each of these modalities have their pros and cons, and their inherent risks and benefits. A large registry of 1010 bAVM patients from The Treatment of Brain Arteriovenous Malformation Study were stratified to surgery, endovascular therapy, or radiosurgery [113]. In total, 229 out of 512 bAVM patients were selected for surgery, with the goal of cure. Surgical cure was achieved in 88% of patients. At the mean time of follow-up, 12% of patients reached the primary safety outcome, with serious adverse events occurring in 21% of patients. Permanent treatment-related complications occurred in 4% of patients, the majority of whom had complications from preoperative embolization; however, one should take care in interpreting these results as there is considerable patient variability with low- and high-grade bAVMs, and the jury is still out regarding whether low-grade, unruptured, bAVMs should be observed rather than subjected to surgery [113]. Similarly, a recent retrospective study of 262 adult patients with unruptured bAVMs, who underwent upfront SRS, reported higher rates of post-treatment hemorrhage, with larger bAVM volumes only among patients with a diffuse nidus compared to those with a compact nidus, exceeding the 2.2% annual rate of post-SRS hemorrhage, suggesting that studying the cytoarchitecture of the bAVM nidus could mitigate post-treatment complications [114]. Finally, recently published consensus guidelines from the ARISE I Consortium (Aneurysm/bAVM/chronic subdural hematoma Roundtable Discussion with Industry and Stroke Experts) recognized the need to improve bAVM characterization, genetic evaluation, and phenotyping in a multidisciplinary manner, with collaborative research efforts to improve outcomes for bAVM patients [4]. As a proper review of these conventional approaches exceeds the scope of this review, we will focus on how investigators have leveraged the understanding of the biological drivers of bAVM formation to run clinical trials using targeted pharmacological therapies. Several of these trials are not specifically aimed at the treatment of bAVMs but are still informative with regard to how pharmacological therapies might be translated into viable strategies for bAVM treatment.

Early trials targeting upstream angiogenic stimulus using tetracycline derivatives, bevacizumab to reduce angiogenic activity, and thalidomide delivered mixed results, with dose-limited patient toxicities to tetracylines [115], with minimal clinical effects on angiogenic activity despite serum reductions in VEGF levels in patients with AVMs treated with bevacizumab [116]. Surprisingly, the drug thalidomide, which was initially prescribed to pregnant women to treat morning sickness in the early 1960s but was discontinued due to teratogenicity, has been found to have anti-angiogenic properties through cytokine inhibition, and the inhibition of TGFα and nitric oxide [117]. In a prospective observational study of 18 patients with severe symptomatic extracranial AVMs treated with thalidomide, all patients experienced reduction in pain, and decreased bleeding and ulceration [118]. Of the twelve patients who stopped treatment due to clinical improvement, eight remained stable, and four had a recurrence of their AVMs within the first year. Adverse events following dose escalation included asthenia, erythroderma, and cerebral infarct (which may have been unrelated to thalidomide drug effect). These initially promising results in treating extracranial AVM patients with thalidomide opens up potential avenues of translation for use in bAVM patients, but further research using preclinical animal models of bAVMs to determine safety would be warranted.

Isolated case reports of using the MEK inhibitor trametinib to target the KRAS/MEK/MAPK pathway as a driver of sporadic AVM formation in the pediatric population with severe extracranial AVMs have reported a good clinical effect [119,120]. One patient also had a spinal intramedullary AVM that responded well to trametinib, with reduced shunting and no reported serious adverse events, opening the door for its potential use in treating intracranial AVMs [121]. A phase II European safety and efficacy trial of trametinib called TRAMAV is currently recruiting adult patients with severe extracranial AVMs (EudraCT: 2019-003573-26). A prospective trial is also currently ongoing in Toronto, Canada, studying the safety and efficacy of MEK inhibition for compassionate use in patients with palliative extra- and intracranial AVMs [74]. These promising results using targeted therapies open avenues of clinical investigation that combine novel and existing treatment modalities to enable more effective treatment outcomes for bAVM patients. We also recognize recently published excellent reviews on the genetic insights, advances in treatments, and emerging therapies for bAVMs to give our readers further references to enrich their learning [122,123,124,125].

## 5. Future Directions

The ability to perform deep sequencing and multi-omic analysis of clinical samples have garnered our ability to gain further insights into the biology of bAVMs. A recent study by Scimone and colleagues conducted differential methylome analysis on ECs from human bAVM samples and compared them to human cerebral microvascular ECs. This study uncovered novel methylated gene loci involved in EC adhesion and crosstalk between EC and vascular smooth muscle cell networks [126]. Not only did this study recapitulate known loci linked to bAVM formation such as *KRAS* and *RBPJ*, but it also identified aberrant methylation patterns at several long non-coding RNA genes targeting transcription factors expressed in neurovascular development, and differential CHG methylated gene clustered in pathways related to EC homeostasis, which point towards more complex mechanisms other than EC dysfunction as a driver of bAVM formation.

A recent study of a single-cell atlas of normal and malformed human brain vasculature by Winkler and colleagues elegantly characterized the immune microenvironment in response to AVM rupture, identifying distinct immune cell clusters including *GPNMB+* monocytes as key players in the depletion of stabilizing smooth muscle cells in AVMs that have bled [11]. They were also able to spatially define cellular and gene expression signatures involving endothelial cell transformations localized to areas around the AVM nidus, and identify clusters of cerebrovascular-derived inflammatory and immune cells associated with sites of hemorrhage, which could lead to novel avenues of therapeutic targeting.

A group from Italy recently reported the use of cell-free DNA next-generation sequencing of liquid biopsy plasma samples to detect mutational burdens in patients with cutaneous AVMs, as a less invasive alternative that avoids morbidities associated with direct lesional sampling [127]. To increase sample sensitivity, they sampled blood from patients at the time of angiography and compared this alongside paired blood from a peripheral blood draw, and were able to detect known mutations in the isolated cell-free DNA sampled from the efferent draining vein. A study by Zenner and colleagues also reported the safety and efficacy of liquid biopsy sampling of peripheral AVMs and venous malformations in detecting driver mutations [128]. Lastly, Winkler and colleagues at the University of San Francisco proposed the “endoluminal biopsy” technique whereby DNA is isolated from the endovascular coil that is placed next to the wall of the AVM vessel lumen prior to a planned endovascular treatment session. The retrieval of the coil following the procedure allowed for the genomic sampling of AVM cells that can be processed for downstream next-generation sequencing, allowing them to identify *KRAS* mutations in four patients with bAVMs [82]. This endoluminal sampling technique could further open avenues of investigation to gain a deeper understanding of the evolving biology of bAVMs and to identify novel targeted therapies that could improve the outcomes of bAVM patients.

Finally, investigators are developing three-dimensional (3D) blood vessel organoids (BVOs) to enable high-throughput screening methods to discover novel treatments for neurovascular disorders. Several 3D vascular organoid models have been developed using human-derived fibroblasts and reprogrammed human pluripotent stem cells (hiPSCs) to mimic different organ systems, including that of the cardiovascular [129], pulmonary [130], gastrointestinal [131], and brain [132]. Oh and colleagues recently utilized 3D BVOs from ECs and compared their gene expression profiles to those of human AVM tissues [133]. Their AVM organoids expressed significantly higher levels of expression of CD31, phalloidin, the angiogenesis-associated gene *FSTL1*, and has-mir-135b-5p, a small RNA related to AVMs. *CSPG4*, a capillary-related gene, exhibited the lowest expression in the 3D AVM organoids. Nikolova and colleagues compiled a comprehensive single-cell genomic atlas of developing hBVOs on the backdrop of genetic and environmental perturbation screens to assess the fate and state landscape of their 3D models of human vasculature [134]. Their hBVOs formed vascular networks of PDGFR-β+, CD31+ ECs, which matured to form vessel lumens. Whilst the goal was to develop diabetic hBVOs that would allow them to study diabetic vasculopathy and not AVM disease, their tunable platform demonstrates the promise in adopting 3D hBVO technology to study neurovascular pathologies. Salewskij and Penninger recently developed self-organizing human capillary blood vessel organoids that recapitulate key processes of vasculogenesis and angiogenesis [135]. One key limitation of these 3D hBVOs is lack of perfusion in vitro, which may be achieved only if transplanted into an animal host for in vivo vascularization and perfusion. This in vivo implantation process unfortunately adds significant experimental costs, precludes high-throughput screening, and introduces potential species-specific effects. Cai and colleagues recently published an elegant study generating 3D vascular network-inspired diffusible (VID) scaffolds of functional midbrain organoids, which were used to test pharmacological responses and neuronal activity changes to fentanyl exposure [136]. These VID scaffolds deliver medium-carrying nutrients, oxygen, and signaling molecules to the organoids with tunable delivery to engineered neural organoids (ENO), leading to reduced apoptosis, stress, and sustained neurogenesis with region-specific functional differentiation. This is in comparison to conventional neural organoids, which developed hypoxic and necrotic cores. Even though this 3D ENO system was not designed with the intent to study the interactions of the neuronal environment around AVMs, the ability to develop 3D vascular networks around functional neural organoids holds promise in their adaptability to study neurovascular disorders. Finally, Kistemaker and colleagues hint at the possibility of the incorporation of hBVOs into a microfluidic chip platform, which could introduce laminar flow and shear stress into the hBVO vasculature to overcome the current limitations of in vitro perfusion, and improve functionality of these hBVO models [137].

## 6. Conclusions

In summary, advances in the understanding of the molecular drivers of non-syndromic and syndromic bAVM formation have allowed for the development of preclinical bAVM models which can be leveraged for translational research. Taken together, these advances hold promise in further characterizing the pathophysiology of bAVMs and offering novel treatment options to decrease the annual hemorrhage rates and subsequent neurological sequelae of bAVM patients.

## Figures and Tables

**Table 1:** Genes implicated in the development of brain arteriovenous malformations and their mechanisms of action.

Study	Genes	Mechanism of Action
Fish et al., 2020 [71]	*MEK-ERK*	Upstream KRAS activation increases MEK kinase activation.
Giarretta et al., 2021 [72]; Shoemaker et al., 2014 [69]	*Shh* *COUP-TFII*	Induced AVM-like properties of vessels. Gli1 and COUP-TFII.
Wang et al., 2023 [73]	*ACVRL1*	Mutation linked to HHT. Found links to sporadic bAVMs.
Mansur and Radovanovic, 2023 [74]; Pérez-Alfayate et al., 2022 [75]	*VEGF*	Upregulated signaling in HHT activates MAPK-ERK pathway. Role in endothelial cell function.
Mansur and Radovanovic, 2023 [74]; Wang et al., 2023 [73]; Pan et al., 2021 [76]; Pérez-Alfayate et al., 2022 [75]; Goss et al., 2019 [77]; Fish et al., 2020 [71]; Nikolaev et al., 2018 [64]	*KRAS*	Increased downstream ERK phosphorylation and angiogenic signaling. Enhanced cell migratory behavior. Somatic KRAS activating mutations: KRAS G12V, KRAS G12D, KRAS G12C, and BRAF. Altered endothelial morphogenesis and growth dynamics.
Murphy et al., 2008 [78]; ZhuGe et al., 2009 [79]; Li et al., 2014 [80]; Pérez-Alfayate et al., 2022 [75];	*NOTCH*	Abnormal gain or loss of NOTCH function. Increased expression of NOTCH-1 and downstream target HES-1 are observed in human bAVM tissue compared to control vessels. *Alk1* knockout mice have decreased Notch signaling. Connects Alk1 and Notch signaling during vascular morphogenesis. Abnormal NOTCH-1 expression in bAVM hemorrhage.
Zhang et al., 2016 [53]; Mansur and Radovanovic, 2023 [74]; Pan et al., 2021 [20]	*Alk1*	Cx3cr1^+^ microglia and Ccr2^+^ macrophages are present in AVM lesions of an *Alk1* deficient mouse model. LOF mutation in HHT patients.
Wang et al., 2023 [73]; Xu et al., 2023 [81]	*TGF-β*	Mutated in ECs, essential for bAVM initiation. Low doses TGF-β stimulate proliferation and migration of ECs through ALK1. High doses of TGF-β result in quiescent endothelium. End-MT in bAVM tissues.
Mansur and Radovanovic, 2023 [74]; Wang et al., 2023 [73]	*RAS-MAPK* *Ex. RASA1*	Mutated in ECs, essential for bAVM initiation. LOF mutation in *RASA1* specifically causes abnormal activation of RAS-MAPK pathway and increases cellular proliferation, growth, differentiation, motility.
Mansur and Radovanovic, 2023 [74]; Pan et al., 2021 [20]; Pérez-Alfayate et al., 2022 [75]	*Endoglin (ENG)*	LOF mutations. ENG is a receptor for TGF-β and BMPs which are predominantly expressed in ECs. LOF undoes BMP/Alk1 signal cascade which suppresses endothelial cell migration and proliferation.
Wang et al., 2023 [73]; Pan et al., 2021 [20]	*SMAD4*	LOF mutation in HHT patients. Linked to juvenile polyposis.
Mansur and Radovanovic, 2023 [74]; Xu et al., 2023 [81]	*BMP9, BMP10*	Mutated in HHT patients. Plays important role in EC function and angiogenesis. BMP9 and BMP10 are probably the natural ligands for the ENG/ALK1 signaling pathway.
Winkler et al., 2022 [82]	*PLVAP, ANGPT2*	Marker of fenestrated endothelium normally confined to developmental angiogenesis, the brain’s circumventricular organs and choroid plexus.
Adhicary et al., 2023 [83]	*Rbpj*	GTPase-mediated cellular function in brain ECs. Deficient expression increased Cdc-42 activity in isolated ECs. Disrupted cell polarity and focal adhesion properties.
Hermanto et al., 2016 [84]; Shoemaker et al., 2014 [69]	*Sox17*	Downstream pathways implicated in bAVM. High expression in thick-walled veins and arteries.

**Abbreviation**: ACVRL1 = activin A receptor-like type 1; ALK1 = activin receptor-like kinase 1; ANGPT2 = angiopoietin 2; AVM = arteriovenous malformation; bAVM = brain arteriovenous malformation; BMP9/10 = bone morphogenetic proteins 9 and 10; BRAF = v-raf murine sarcoma viral oncogene homolog B1; Cdc42 = cell division control protein 42 homolog; Cx3cr1 = C-X3-C motif chemokine receptor 1; Ccr2 = C-C motif chemokine receptor 2; COUP-TFII = chicken ovalbumin upstream promoter transcription factor II; ECs = endothelial cells; ENG = endoglin; ERK = extracellular signal-regulated kinase; HES1 = hairy and enhancer of split 1; HHT = hereditary hemorrhagic telangiectasia; KRAS = Kirsten rat sarcoma viral oncogene homolog; LOF = loss of function; MAPK = mitogen-activated protein kinase; MEK = MAPK/ERK kinase; NOTCH = neurogenic locus notch homolog protein; PLVAP = plasmalemma vesicle-associated protein; RAS = rat sarcoma viral oncogene homolog; RASA1 = RAS p21 protein activator 1; RAS-MAPK = rat sarcoma–mitogen-activated protein kinase; Shh = sonic hedgehog; SMAD = mothers against decapentaplegic homolog; Sox17 = SRY-box transcription factor 17; TGF-β = transforming growth factor beta; and VEGF = vascular endothelial growth factor.

**Table 2:** Cell types associated with the brain arteriovenous malformation microenvironment and their molecular roles.

Studies	Cell Type	Role in AVM Biology
Winkler et al., 2022 [11]; Wang et al., 2023 [73]	Macrophages	Perivascular macrophages (28.3% of bAVMs), IBA1^+^P2RY12^−^ MΦ. Significantly increased in bAVM tissue.
Winkler et al., 2022 [11]	Monocytes	*AIF1^+^*-*P2RY12^−^* monocytes over-represented in ruptured bAVMs.
Winkler et al., 2022 [11]	Microglia	Discrete areas have numerous IBA1+ PSRY12+ monocytes.
Wang et al., 2023 [73]	Neutrophils	Significantly increased in bAVM tissue.
Winkler et al., 2022 [11]; Shabani et al., 2022 [85]	T lymphocytes	CD4+, CD8+, Tregs found in immune cell clusters associated with cerebrovasculature. Predominant detection in unruptured bAVM tissue.
Winkler et al., 2022 [11]	Natural killer cells	Found in immune cell clusters associated with cerebrovasculature.
Winkler et al., 2022 [11]	Plasmacytoid Dendritic Cells	Found in immune cell clusters associated with cerebrovasculature.
Tu et al., 2025 [86]; Shabani et al., 2022 [85]; Thomas et al., 2021 [87]	Astrocytes	Promotion of angiogenesis and vascular instability (hemorrhagic risk). Aberrant expressions of ALDH1A2 and CYR61 in abnormal neighboring astrocytes.
Winkler et al., 2022 [11]; Mansur and Radovanovic, 2023 [74]; Nikolaev et al., 2018 [64]; Shabani et al., 2022 [85]	Endothelial Cells	*CLDN5^+^* within bAVM cell population. Clusters with suppressed venule and capillary cell identities. Alk1, Eng, and SMAD transcription factors work to suppress migration. VEGF and ET-1. KRAS mutations.
Winkler et al., 2022 [11]	Fibromyocytes	*CCL19*^+^ within bAVM cell population.
Winkler et al., 2022 [11]	Smooth Muscle Cells	*TAGLN^+^* within bAVM cell population.
Winkler et al., 2022 [11]	Perivascular Fibroblasts	*COL1A2^+^* within bAVM cell population.
Winkler et al., 2022 [11]; Pan et al., 2021 [76]; Nakisli et al., 2023 [88]; Shabani et al., 2022 [85]	Mural Cells	*KCNJ8+* Pericyte number and coverage reduced. PDGF-B/PDGFR-disruption. Notch signaling pathway. BMP/ALK/SMAD pathway. RAS/MAPK pathway.
Winkler et al., 2022 [11]	Perivascular Fibroblasts	*DCN* ^+^ *APOD* ^+^
Shoemaker et al., 2020 [70]; Xu et al., 2023 [81]	Mesenchymal Cells	The result of endothelial–mesenchymal transition signaling within bAVMs.

**Abbreviations**: ALDH1A2 = aldehyde dehydrogenase 1 family member A2; ANGPT2 = angiopoietin 2; APO D = apolipoprotein D; AVM = arteriovenous malformation; bAVM = brain arteriovenous malformation; BMP = bone morphogenetic protein; CD4+ = cluster of differentiation 4 positive; CD8+ = cluster of differentiation 8 positive; CLDN5 = claudin-5; COL1A2 = collagen type I alpha 2 chain; CCL19 = C-C motif chemokine ligand 19; CYR61 = cysteine-rich angiogenic inducer 61; DCN = decorin; ECs = endothelial cells; ENG = endoglin; ET-1 = endothelin-1; HHT = hereditary hemorrhagic telangiectasia; IBA1 = ionized calcium-binding adaptor molecule 1; KRAS = Kirsten rat sarcoma viral oncogene homolog; MAPK = mitogen-activated protein kinase; MΦ = macrophage; PDGF-B = platelet-derived growth factor subunit B; PDGFR = platelet-derived growth factor receptor; P2RY12 = purinergic receptor P2Y12; SMAD = mothers against decapentaplegic homolog; TAGLN = transgelin; TGF-β = transforming growth factor beta; and VEGF = vascular endothelial growth factor.

**Table 3:** Animal models of brain arteriovenous malformations.

Animal Model	Features
*Eng^f/f^* mice [96]	- Ad-Cre-treated brains have *Eng*-null ECs. - VEGF induced more severe vascular dyplasia in Ad-Cre-treated brains of *Eng^f/f^* mice compared with *Eng^+/−^* mice.
*Eng* or *Alk1* conditional knockout mice [16,112]	- Inducible conditional knockout of *Eng* or *Alk1,* specifically in brain ECs but not in pericytes or macrophages. - Leads to focal angiogenic stimulation in the brains of mice.
*Eng* or *Alk1* conditional knockout mice [100]	- Showed that over-expression of *Alk1* can rescue the AVM phenotype via normalizing expression of Notch and Smad pathways gene in *Eng*-deficient ECs.
*Kras^G12D^* or *Kras^G12V^* transgenic mice [71,110]	- Brain EC-specific *Kras* G12D or G12V gain-of-function mutations in mice lead to formation of bAVMs. - Adenovirus-associated viral vector infection of *Kras* G12D into brain ECs of mice promotes bAVM formation via activation of the mitogen-activated protein kinase (MAPK) pathway. - 50% of mice developed bAVM which may not necessarily require physiological angiogenesis during early development.
*Int3* transgenic mice [78]	- Constitutive expression of *int3*, the murine homolog of *Notch4*, in brain ECs of tetracycline-regulated transgenic mice leads to formation of dilated and tangled brain vessels that hemorrhage. - Repression of endothelial *int3* expression in P20- or P21-day-old mice reverses neurological deficits.
*Kras^G12D^* zebrafish [71]	- Constitutively active *Kras* G12D into ECs. - 50% of zebrafish developed AV shunts. - NOT completely representative of human bAVMs. - Established shunts were reversed by pharmacological MEK inhibition but refractory to PI3K inhibition.

**Abbreviations**: Ad-Cre = adenovirus–Cre recombinase; ALK1 = activin receptor-like kinase 1; AVM = arteriovenous malformation; bAVM = brain arteriovenous malformation; ECs = endothelial cells; ENG = endoglin; HHT = hereditary hemorrhagic telangiectasia; Int3 = intracellular domain of Notch4 (murine homolog of Notch4); KRAS = Kirsten rat sarcoma viral oncogene homolog; MAPK = mitogen-activated protein kinase; P20/P21 = postnatal day 20/postnatal day 21; SMAD = mothers against decapentaplegic homolog; and VEGF = vascular endothelial growth factor.

## Data Availability

No new data were created or analyzed in this study.
